# Supplementary material for: 3D Quantification of Viral Transduction Efficiency in Living Human Retinal Organoids
Source: Small Methods. 2025 Jun 12;10(2):2401050. doi: 10.1002/smtd.202401050 (PMC12825330; doi:10.1002/smtd.202401050)
Supplement: Supplementary file 1 — Supporting Information [file SMTD-10-2401050-s002.pdf]

3D Quantification of Viral Transduction Efficiency in Living Human Retinal Organoids

Teresa S. Rogler[1,2,3], Katja A. Salbaum[2], Achim T. Brinkop[1,2], Selina M. Sonntag[2], Rebecca James[2], Elijah R. Shelton[2], Alina Thielen[2], Roland Rose[2], Sabrina Babutzka[3,6], Thomas Klopstock[4,5,6], Stylianos Michalakis[3,7], Friedhelm Serwane[1,2,3,4]

[1]Institute of Biophysics, Ulm University, Ulm, Germany

[2]Faculty of Physics and Center for NanoScience (CeNS), LMU, Munich, Germany

[3]Graduate School of Systemic Neuroscience (GSN), Munich, Germany

[4]Munich Cluster for Systems Neurology (SyNergy), Munich, Germany

[5]Friedrich Baur Institute at the Department of Neurology, University Hospital, LMU Munich, Germany

[6]German Center for Neurodegenerative Diseases (DZNE), Munich, Germany

[7]Department of Ophthalmology, LMU Hospital, LMU Munich, Munich, Germany

Supplementary Information

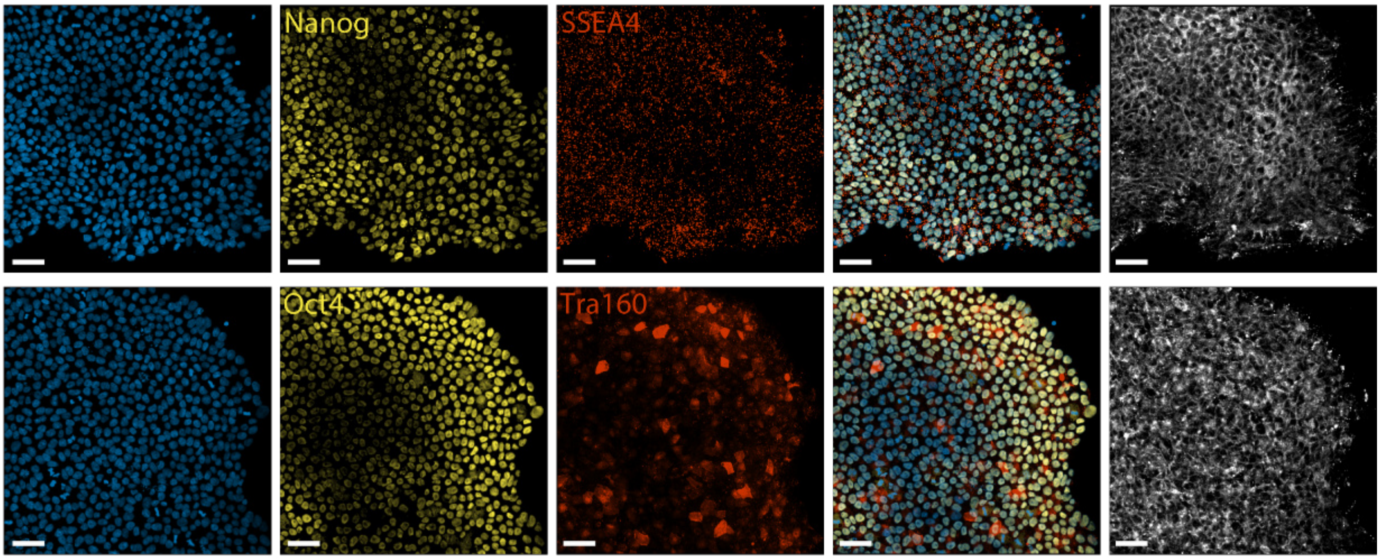

Supplementary Figure S1. Confocal images of pluripotency staining of iPS(IMR90)-4p37. Primary antibodies indicated in yellow and red, blue: DAPI, white: WGA-CF membrane stain. Scale: 50 microns.

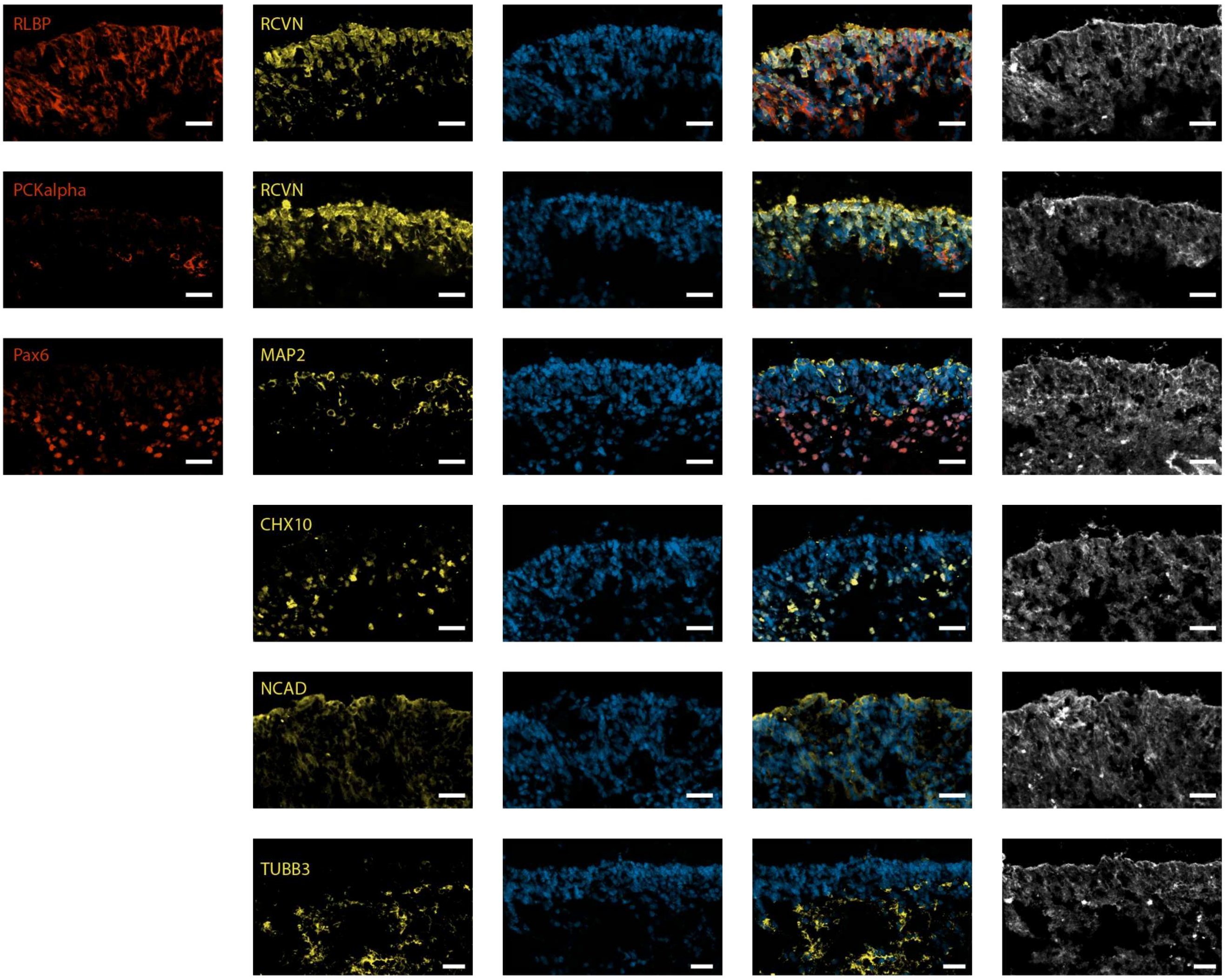

Supplementary Figure S2. Confocal images of immunofluorescence staining DIV227 human retinal organoids. Primary antibodies indicated in red and yellow, blue: DAPI, white: WGA-CF membrane stain. Scale bar: 30 microns. The markers were selected to highlight retina typical markers: RLBP (Retinaldehyde binding protein 1; müller glia cells), RCVN (recoverin; photoreceptor marker), PKCalpha (protein kinase C alpha; rod bipolar cells), Pax6 (paired box 6; neuronal progenitors), Map2 (microtubule associated protein 2; neuronal marker), CHX10 (visual system homeobox 2; retinal progenitor cells, bipolar cells), NCAD (N-cadherin; cell-cell adhesion, role in retina lamination) , TUBB3 (tubulin beta 3; microtubule, axone)

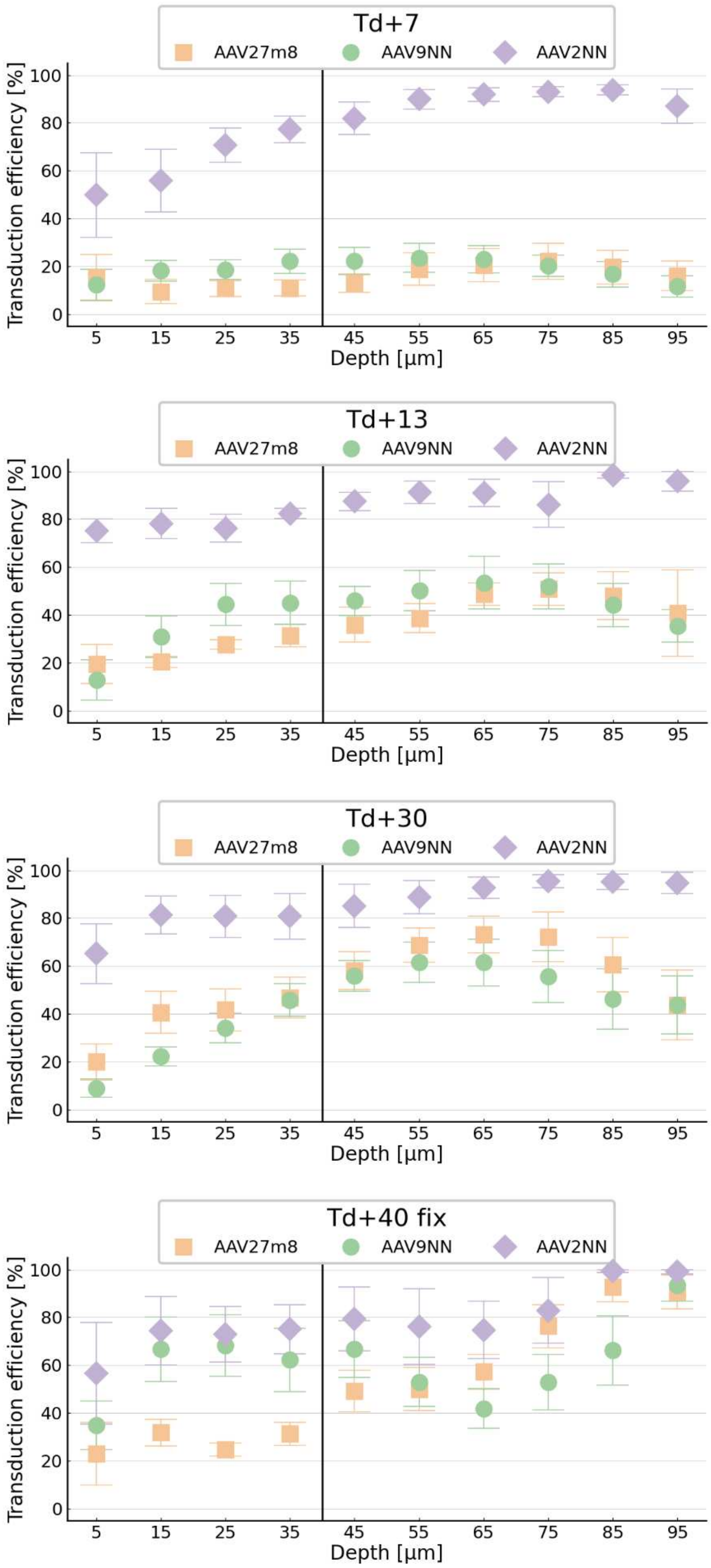

Supplementary Figure S3. Additional data for Figure 6A. Spatially resolved quantification of viral vector transduction efficiency in living retinal organoids at all timepoints. Transductions efficiency was binned over 10 µm deep z-slices. Error bars indicate the standard error of the mean (SEM).

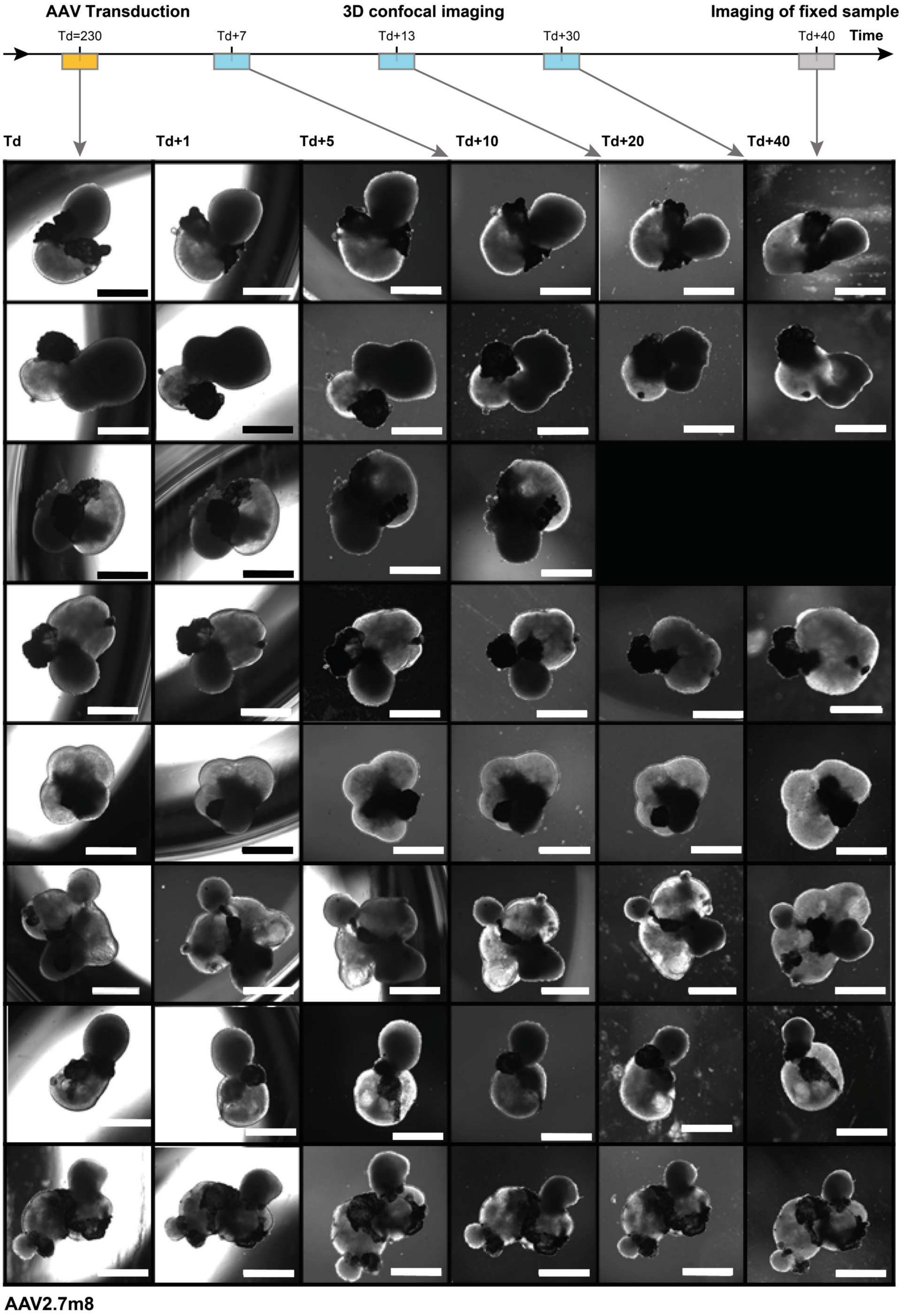

Supplementary Figure S4. Brightfield images of 8 human retinal organoids transduced with AAV2.7m8 and imaged at six time points after transduction (Td), related to Figure 4. Organoids from transduction experiment T1-8 from top to bottom. T1 was transduced at DIV227, T8 at DIV235. Scale bars: 500 microns.

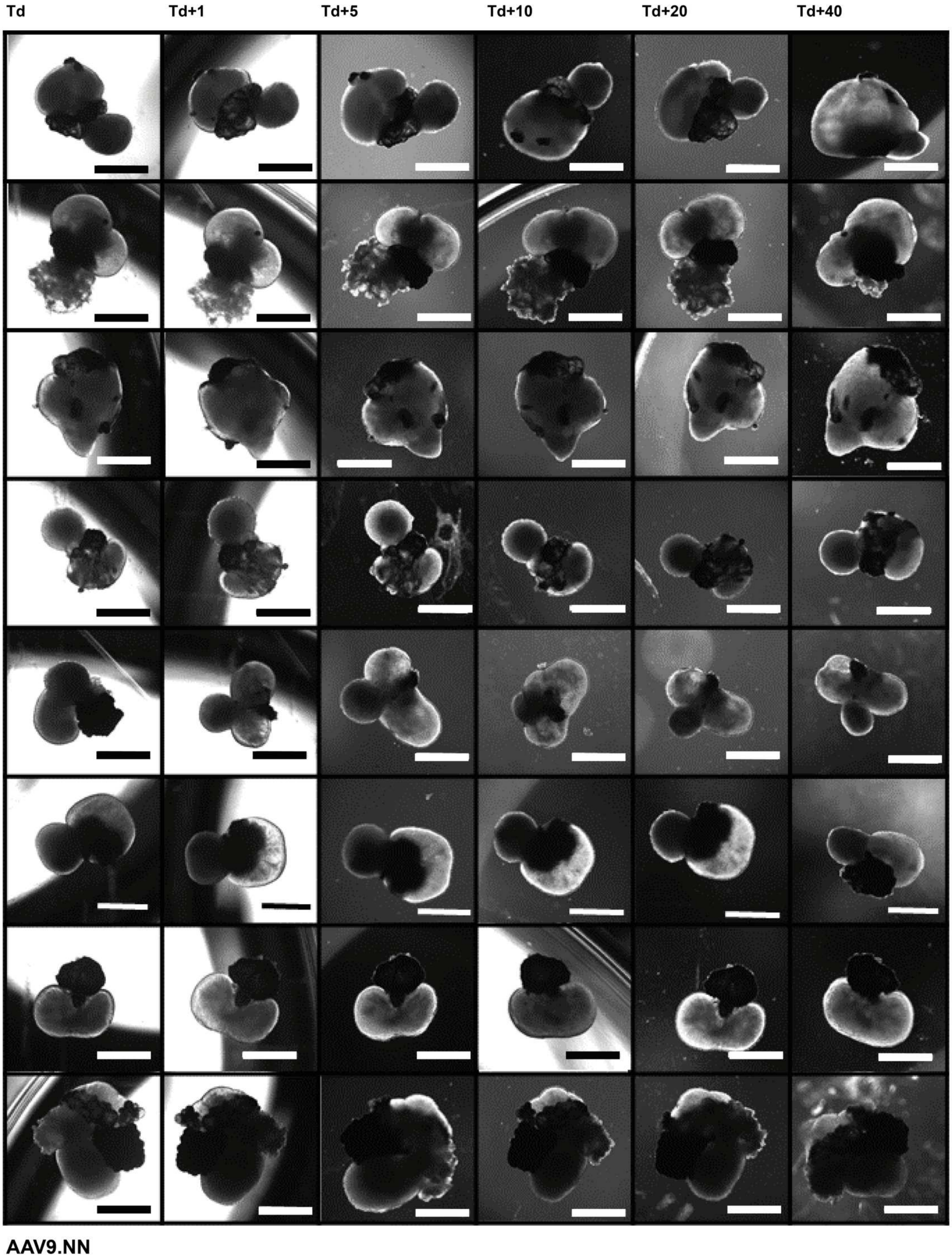

Supplementary Figure S5. Brightfield images of 8 human retinal organoids transduced with AAV9.NN and imaged at six time points after transduction (Td), related to Figure 4. Organoids from transduction experiment T1-8 from top to bottom. T1 was transduced at DIV227, T8 at DIV235. Scale bars: 500 microns.

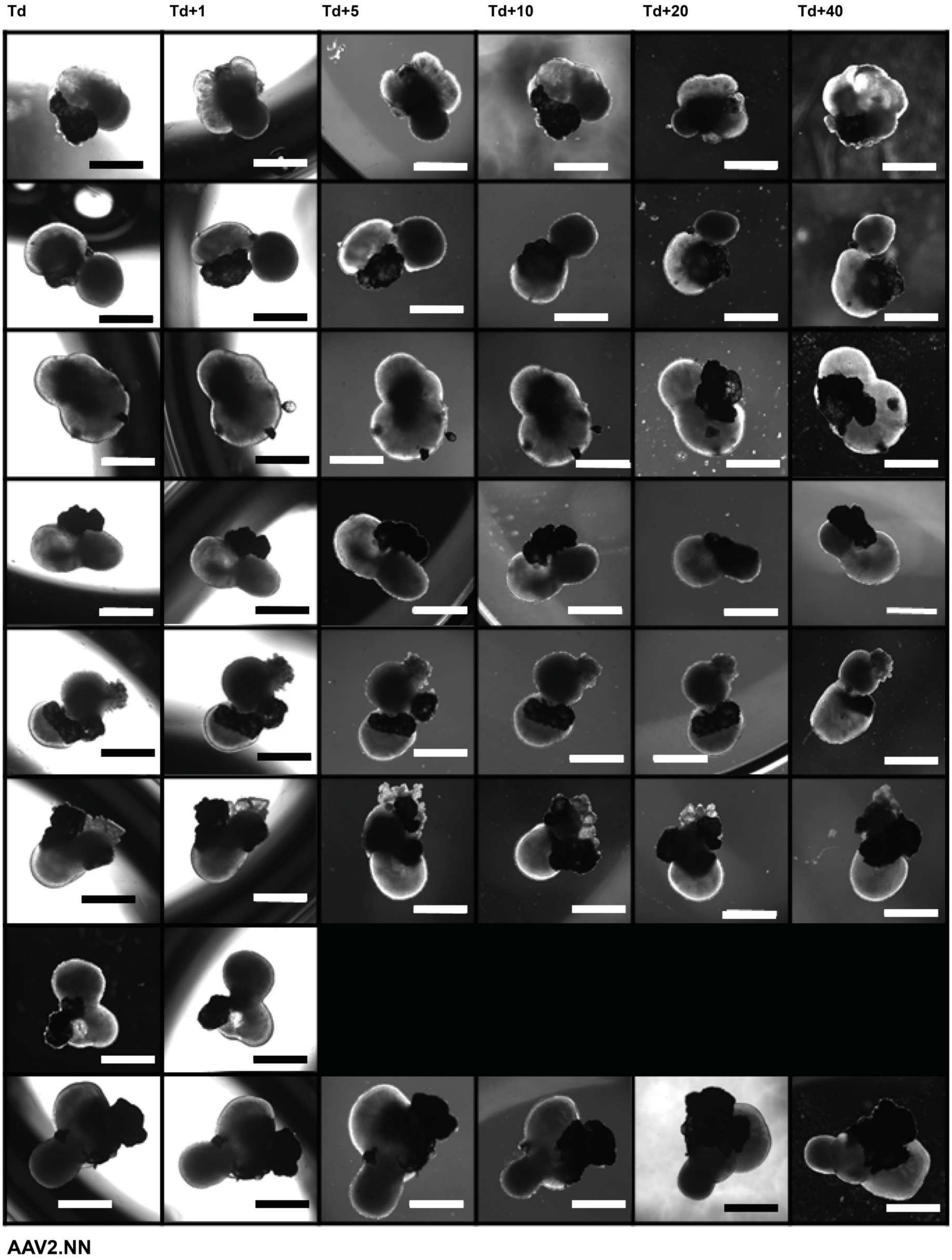

Supplementary Figure S6. Brightfield images of 8 human retinal organoids transduced with AAV2.NN and imaged at six time points after transduction (Td), related to Figure 4. Organoids from transduction experiment T1-8 from top to bottom. T1 was transduced at DIV227, T8 at DIV235. Scale bars: 500 microns.

Supplementary Table S1. Reagents used for live imaging, staining of cryosectioned organoids, and pluripotency staining of iPSCs.

Antibody	Manufacturer	Product number	Dilution (Concentration)
Recoverin	Sigma	AB5585	500
RLBP1/CRALBP	abcam	AB15051	100
RBPMS	PhosphoSolutions	1830-RBPMS	100
RBPMS-thermo	Thermo Fisher Scientific	PA5-31231	100
Tuj1	Biolegend	801213	100
PCKalpha	Santa Cruz Biotechnology	sc-8393	100
n-Cadherin	Thermo Fisher Scientific	PA5-29570	200
Rx	Santa Cruz Biotechnology	sc-271889	200
MAP2 aves	Aves labs	MAP-0020	500
Pax6	GeneTex	GTX113241	100
ZO-1	Thermo Fisher Scientific	40-2200	100
CHX10	Thermo Fisher Scientific	PA1-12565	200
Nanog	Gift from D. Paquet		500
Oct4	Gift from D. Paquet		500
SSEA4	Gift from D. Paquet		100
Tra160	Gift from D. Paquet		500
DAPI	Invitrogen	D1306	(0.1 µg/ml)
WGA CF 555	Biotium	29076	1000
Goat anti-Rabbit IgG (H+L) Cross-Adsorbed Secondary Antibody, Alexa Fluor 488	Invitrogen	A-11008	500
Goat anti-Chicken IgY (H+L) Secondary Antibody, Alexa Fluor 488	Invitrogen	A-11039	500
Goat anti-Mouse IgG (H+L) Cross-Adsorbed Secondary Antibody, Alexa Flour 647	Invitrogen	A-21235	500
Goat anti-Rabbit IgG (H+L) Cross-Adsorbed Secondary Antibody, Alexa Fluor 647	Invitrogen	A-21244	500

Donkey anti-Sheep IgG (H+L) Cross-Adsorbed Secondary Antibody, Alexa Fluor 647	Invitrogen	A-21448	500
NucSpot Live 650 Nuclear Stain	Biotium	40082-T	250
Cellbrite steady 550 membrane	Biotium	30107-T	1000

Supplementary Table S2. Data for Figure 4G and H. Manual counting of transduced cells for different serotypes. Three image layers of z-stacks for different AAV serotypes were manually annotated by human annotators (layer 100 (25 µm), layer 200 (50 µm) and layer 300 (75 µm). The total number of cells and the number of not transduced cells was counted by examining the membrane dye channel (CellBrite550). The number of transduced cells were then calculated by subtracting the non-transduced from the transduced cells. This data can was then averaged over the layers in Figure 4G and H.

Serotype	Layer	#Cells	#Transduced	#Not transduced	Transduction efficiency [%]
**Human annotator 1**					
AAV2.NN	100	1254	989	265	79
	200	1391	1130	261	81
	300	648	451	197	70
AAV9.NN	100	1776	364	1412	20
	200	1666	406	1260	24
	300	797	226	571	28
AAV2.7m8	100	915	116	799	13
	200	919	99	820	11
	300	627	124	503	20
**Human annotator 2**					
AAV2.NN	100	801	631	170	79
	200	445	375	70	84
	300	125	69	56	55
**Human annotator 3**					
AAV2.NN	100	1288	754	534	59
	200	926	633	293	68
	300	362	290	72	80

Supplementary Table S3. Data for Figure 4G. Automated transduction efficiency as a comparison for manual counting in Supplementary Figure 2. Three different thresholds were assessed to determine which one is best to be used in the automatized analysis pipeline. One image set of one organoid transduced each with a different serotype was manually annotated and the calculated transduction efficiency was averaged over the layers. The result was compared to the automated results in the same image stacks. The table shows the transduction efficiency resulting from the pipeline at different thresholds and the averaged transduction efficiency calculated from the manual counting averaged over the layers and its standard deviation.

	Transduction efficiency [%]				
**Serotype**	**Threshold 30 %**	**Threshold 40 %**	**Threshold 50 %**	**Human annotator**	**Standard deviation human annotator**
AAV2.NN	89	84	78	77	6
AAV9.NN	34	24	16	24	4
AAV2.7m8	20	15	12	14	5

Supplementary Table S4. Data for Figure 5C. Serotype transduction efficiency for individual organoids at different timepoints after transduction. The organoid symbols refer to the symbols used for organoid identification in Figure 5C. DIV (day in vitro) indicates the age of the organoid at the timepoint it was image. The transduction efficiency was averaged if the number of areas imaged in the same organoid was bigger than one.

Serotype	Organoid symbol	Timepoint	DIV	Transduction efficiency (%)	Number of areas
AAV2.7m8	circle	Td+13	DIV244	38	1
AAV2.7m8	circle	Td+30	DIV257	62	1
AAV2.7m8	circle	Td+40	DIV267	35	1
AAV2.7m8	square	Td+7	DIV235	31	1
AAV2.7m8	square	Td+30	DIV258	83	1
AAV2.7m8	square	Td+40	DIV268	66	1
AAV2.7m8	triangle pointing left	Td+7	DIV236	5	1
AAV2.7m8	star	Td+7	DIV237	2	1
AAV2.7m8	star	Td+30	DIV260	64	2
AAV2.7m8	star	Td+40	DIV270	27	1
AAV2.7m8	cross	Td+7	DIV238	14	2
AAV2.7m8	cross	Td+30	DIV261	20	1
AAV2.7m8	cross	Td+40	DIV271	37	2
AAV2.7m8	pentagon	Td+7	DIV239	26	1
AAV2.7m8	pentagon	Td+13	DIV245	42	1
AAV2.7m8	pentagon	Td+30	DIV262	56	2
AAV2.7m8	pentagon	Td+40	DIV272	44	2
AAV2.7m8	triangle pointing right	Td+7	DIV240	14	3
AAV2.7m8	triangle pointing right	Td+13	DIV246	36	2
AAV2.7m8	triangle pointing right	Td+30	DIV263	41	1
AAV2.7m8	diamond	Td+7	DIV241	8	2
AAV2.7m8	diamond	Td+13	DIV247	28	2
AAV2.7m8	diamond	Td+30	DIV264	38	2
AAV2.7m8	diamond	Td+40	DIV274	38	2
AAV2.NN	circle	Td+7	DIV234	99	1
AAV2.NN	circle	Td+13	DIV244	78	2
AAV2.NN	circle	Td+30	DIV257	98	1
AAV2.NN	circle	Td+40	DIV267	90	1
AAV2.NN	square	Td+7	DIV235	82	1
AAV2.NN	square	Td+30	DIV258	98	1
AAV2.NN	square	Td+40	DIV268	95	1
AAV2.NN	triangle pointing left	Td+7	DIV236	69	2
AAV2.NN	triangle pointing left	Td+30	DIV259	96	2
AAV2.NN	triangle pointing left	Td+40	DIV269	62	2
AAV2.NN	star	Td+7	DIV237	90	1
AAV2.NN	star	Td+30	DIV260	79	2
AAV2.NN	cross	Td+7	DIV238	90	1
AAV2.NN	cross	Td+30	DIV261	51	2
AAV2.NN	cross	Td+40	DIV271	38	1
AAV2.NN	pentagon	Td+7	DIV239	84	1
AAV2.NN	pentagon	Td+13	DIV245	88	1
AAV2.NN	pentagon	Td+30	DIV262	99	1
AAV2.NN	pentagon	Td+40	DIV272	98	1
AAV2.NN	diamond	Td+7	DIV241	57	2
AAV2.NN	diamond	Td+13	DIV247	79	3
AAV2.NN	diamond	Td+30	DIV264	86	1
AAV9.NN	circle	Td+7	DIV234	24	1

AAV9.NN	circle	Td+13	DIV244	40	2
AAV9.NN	circle	Td+30	DIV257	26	1
AAV9.NN	circle	Td+40	DIV267	84	2
AAV9.NN	square	Td+7	DIV235	35	1
AAV9.NN	square	Td+30	DIV258	66	1
AAV9.NN	square	Td+40	DIV268	79	1
AAV9.NN	triangle pointing left	Td+7	DIV236	15	2
AAV9.NN	triangle pointing left	Td+30	DIV259	55	1
AAV9.NN	triangle pointing left	Td+40	DIV269	26	1
AAV9.NN	star	Td+7	DIV237	7	1
AAV9.NN	star	Td+40	DIV270	30	1
AAV9.NN	cross	Td+7	DIV238	11	2
AAV9.NN	cross	Td+30	DIV261	27	1
AAV9.NN	cross	Td+40	DIV271	83	1
AAV9.NN	pentagon	Td+7	DIV239	33	1
AAV9.NN	pentagon	Td+13	DIV245	35	2
AAV9.NN	pentagon	Td+30	DIV262	58	1
AAV9.NN	pentagon	Td+40	DIV272	70	1
AAV9.NN	triangle pointing right	Td+7	DIV240	11	2
AAV9.NN	triangle pointing right	Td+13	DIV246	37	2
AAV9.NN	diamond	Td+13	DIV247	62	1
AAV9.NN	diamond	Td+30	DIV264	49	1

Supplementary Table S5. Data for figure 5C. Mean serotype transduction efficiency at different timepoints after transduction.

Serotype	Timepoint	Mean transduction efficiency [%]	Standard error of the mean [%]
AAV2.7m8	Td+7	14	5
AAV2.7m8	Td+13	36	3
AAV2.7m8	Td+30	52	8
AAV2.7m8	Td+40	41	6
AAV9.NN	Td+7	19	5
AAV9.NN	Td+13	43	7
AAV9.NN	Td+30	47	7
AAV9.NN	Td+40	62	12
AAV2NN	Td+7	82	6
AAV2NN	Td+13	82	4
AAV2NN	Td+30	87	7
AAV2NN	Td+40	77	12

Supplementary Table S6. Data for figure 6A. Mean serotype transduction efficiency at different depths on Td+7.

Serotype	Bin [µm]	Mean transduction efficiency [%]	Standard error of the mean [%]
AAV2.7m8	0-10	15	10
AAV2.7m8	10-20	9	6
AAV2.7m8	20-30	11	4
AAV2.7m8	30-40	11	4
AAV2.7m8	40-50	13	4
AAV2.7m8	50-60	19	7
AAV2.7m8	60-70	20	7
AAV2.7m8	70-80	22	8

AAV2.7m8	80-90	20	8
AAV2.7m8	90-100	16	7
AAV9.NN	0-10	12	7
AAV9.NN	10-20	18	5
AAV9.NN	20-30	19	5
AAV9.NN	30-40	22	6
AAV9.NN	40-50	22	6
AAV9.NN	50-60	24	6
AAV9.NN	60-70	23	6
AAV9.NN	70-80	20	5
AAV9.NN	80-90	17	6
AAV9.NN	90-100	11	5
AAV2NN	0-10	50	18
AAV2NN	10-20	56	14
AAV2NN	20-30	71	8
AAV2NN	30-40	77	6
AAV2NN	40-50	82	7
AAV2NN	50-60	90	5
AAV2NN	60-70	91.9	2.9
AAV2NN	70-80	93.1	2.1
AAV2NN	80-90	93.8	2.2
AAV2NN	90-100	87	8

Supplementary Movie S1: Confocal image and segmentation of a 226 days old human retinal organoid transduced with AAV2.7m8-egfp**.** The cytoplasmic eGFP is displayed in cyan, the stained nuclei in magenta and the nuclei segmentation in grey-scale. The ellipsoidal approximation of nuclei belonging to transduced cells is displayed in a color encoding the z position (spectral gradient) in the retina.
